# Factors related to survival time in dogs with pulmonary hypertension secondary to degenerative mitral valve disease stage C

**DOI:** 10.1080/23144599.2022.2067630

**Published:** 2022-04-29

**Authors:** Jutamas Udomkiattikul, Noppasorn Kirdratanasak, Panatsada Siritianwanitchakul, Wasaporn Worapunyaanun, Sirilak Disatian Surachetpong

**Affiliations:** Department of Veterinary Medicine, Faculty of Veterinary Science, Chulalongkorn University,Pathumwan, Thailand

**Keywords:** Dogs, degenerative mitral valve disease, pulmonary hypertension, survival time

## Abstract

Pulmonary hypertension (PH) is a common complication in dogs with degenerative mitral valve disease (DMVD). The aim of this study was to determine the survival time and to determine factors related to the survival time of dogs with PH secondary to DMVD stage C. A retrospective study was conducted in 37 dogs with PH secondary to DMVD stage C to analyse the median survival time and associated factors such as age, sex, breed, body weight, the presence of syncope, heart rate, systolic blood pressure, manifestation of congestive heart failure (CHF), vertebral heart score (VHS), the presence of left or right heart enlargement, probability of PH and medication. Data were analysed using the Log-Rank test and plotted with the Kaplan-Meier curve. The results showed that the median survival time of dogs with PH secondary to DMVD stage C was 368 days, and factors that shortened the median survival time of dogs with PH secondary to DMVD stage C were mixed breeds, VHS > 11.5, the presence of right heart enlargement, ascites and high probability of PH. Factors associated with an increased hazard of death were mixed breed dogs, dogs with right heart enlargement and ascites. These findings may be useful for the prognosis and management of dogs with PH secondary to DMVD stage C.

## 1. Introduction

Degenerative mitral valve disease (DMVD), the most common acquired heart disease in small-breed dogs, is caused by degeneration of the heart valves, leading to systolic mitral valve regurgitation (MR). Common adverse outcomes of DMVD include congestive heart failure (CHF) and pulmonary hypertension (PH) [1,2]. In DMVD, PH may be caused by an initial increase in left ventricular filling pressure to the pulmonary capillaries, which can be reversed in the early stage of the disease. However, in the final stage, when the pulmonary vascular remodelling begins, PH becomes irreversible [3].

Based on the American College of Veterinary Internal Medicine (ACVIM) consensus guidelines for the diagnosis and management of DMVD in dogs [2], DMVD is classified into four stages, depending on cardiac morphology and clinical signs, including stage A, B, C and D. The present study focused on dogs with PH secondary to DMVD stage C with clinical signs of heart failure. The diagnosis of dogs with DMVD stage C is based on clinical findings of heart failure including pulmonary oedema assessed by thoracic radiography and echocardiographic assessment of cardiac remodelling [2].

The development of PH may be caused by increased pulmonary venous pressure, increased pulmonary blood flow and increased pulmonary vascular resistance. The presence of PH has been classified depending on the probability of PH, which is categorized as low, intermediate and high, based on the peak velocity of tricuspid regurgitation (TRV) and the number of different anatomic sites of echocardiographic signs of PH, the severity of PH, and the cause of PH. Clinical findings of PH in dogs included syncope, respiratory distress at rest, exercise intolerance and ascites [4]. One study reported that the detection of PH in dogs with DMVD stage B2 and C is associated with poor prognosis [5].

Few studies have reported the survival time of DMVD dogs at various stages [6,7]. A previous study showed an association of cardiac death with heart murmurs, the ratio of left atrial dimension to the aortic annulus dimension (LA:Ao) > 1.7 and the peak velocity of early diastolic transmitral flow (MV E velocity) > 1.2 [6]. Another previous study showed that dyspnoea, pulmonary oedema and vertebral heart score (VHS) > 10.5 were associated with a higher possibility of death [7]. The presence of PH in DMVD dogs was associated with a shorter survival time [5].

As mentioned previously, clinical findings and median survival time may worsen in dogs affected by PH secondary to DMVD. However, there is no study reporting the factors related to the median survival time of PH dogs affected secondary to DMVD stage C in Thailand. This study aimed to determine the median survival time, prognostic indicators and treatment strategies in dogs with PH secondary to DMVD.

## 2. Materials and methods

### 2.1. Data of dogs

Electronic medical records from the Small Animal Teaching Hospital, Faculty of Veterinary Science, Chulalongkorn University from August 2015 to June 2021 were collected. Ethical approval was not required for this study because of its retrospective design. The database assessment was approved by the Chulalongkorn University Small Animal Teaching Hospital Board committees.

Data of thirty-seven dogs affected DMVD stage C with PH were recruited to the study. The inclusion criteria were dogs diagnosed with PH secondary to DMVD stage C and died within June 2021. Diagnostic criteria for DMVD stage C consisted of an echocardiographic examination with the presence of LA:Ao ≥ 1.6 and a normalized left ventricular internal diameter in the diastole (LVIDDN) ≥ 1.7. All dogs had to have past or present clinical signs of left-sided heart failure and evidence of pulmonary oedema assessed by thoracic radiography. Diagnostic criteria for the probability of PH consisted of a TRV > 3 m/s^2^ or a pressure gradient between the right ventricle and right atrium > 46 mmHg, and anatomic sites of the echocardiographic signs of PH [4]. Telephone interviews were conducted to confirm the death or alive status of dogs that the status or date of death could not be reported by the end of June 2021. Dogs that were alive after this date or whose death or alive status could not be confirmed were excluded from the study.

### 2.2. Clinical evaluation

Left-sided heart failure clinical findings were tachypnoea, restlessness, dyspnoea, cough, exercise intolerance, cyanosis and radiographic findings of pulmonary oedema. Pleural effusion and ascites, the most important clinical findings of right-sided heart failure were also documented. Syncope, a common clinical sign of PH, was also noted.

Data of blood pressure measured by using an ultrasonic Doppler device (model 811; Parks Medical Electronics, Beaverton, Oregon, USA) were recorded. The pressure measurement was performed according to the ACVIM Consensus Guidelines [8]. The cuff size of approximately 40% of limb circumferences was used. Blood pressure was measured at left forelimb around carpal joint area in sternal recumbent position. Blood pressure measurement was performed at least 5 times in each dog in a quiet room to reduce stress induced situational hypertension.

**Figure 1.**
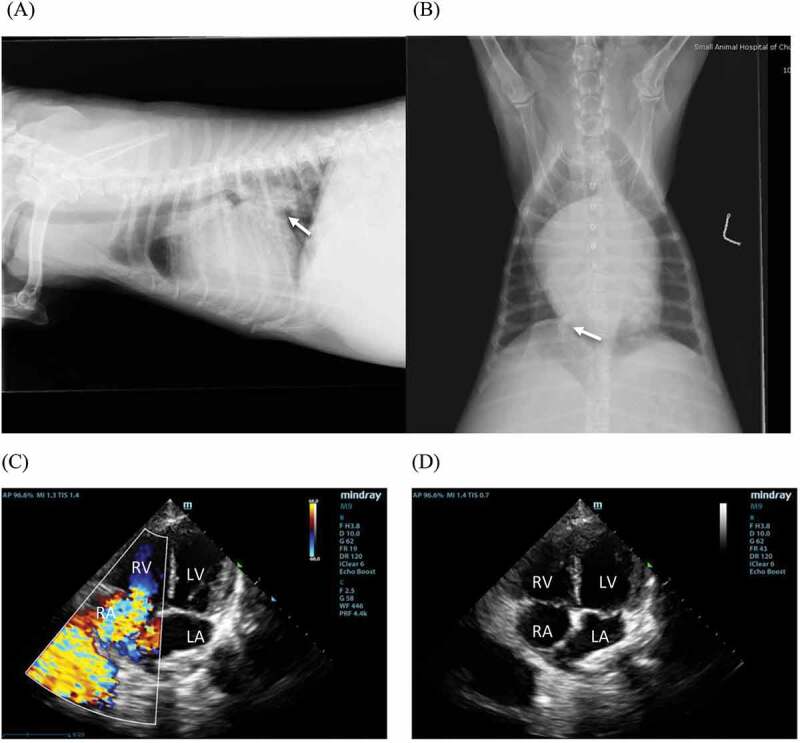
The radiography of a 10-year-old female poodle dog with pulmonary hypertension (PH) secondary to degenerative mitral valve disease (DMVD). A, the right lateral radiograph shows an enlarged cardiac silhouette with left atrial enlargement (arrow) and caudal lung lobe infiltration. B, the ventrodorsal radiograph shows right heart enlargement and pulmonary artery congestion (arrow). The echocardiography of a 10-year-old male mixed breed dog with PH due to DMVD. C, D, the left apical four chamber view echocardiography shows tricuspid regurgitant flow on colour Doppler on two-dimensional (2D) echocardiography and right atrial (RA) and right ventricular enlargement on 2D echocardiography.

Two views thoracic radiographs (right lateral and ventrodorsal views) were obtained and evaluated. Vertebral heart score was measured according to the method described in a previous study [9]. The right heart enlargement was determined by increased sternal contact in the lateral view or an inverted D-shaped cardiac silhouette in the ventrodorsal view (Figure 1).

Echocardiography was performed by an experienced investigator (SS) using an ultrasound machine (M9, Mindray, China) with multifrequency 4–12 MHz phased-array transducers. Echocardiography was performed without sedation. The right parasternal long-axis view was used to determine the characteristics of the mitral and tricuspid valves. Left ventricular internal diameters in systole (LVIDS) and diastole (LVIDN) were measured in the right parasternal four- chamber view with M-mode echocardiography. M-mode echocardiographic values were normalized by using the Cornell’s allometric scaling method [10]. The percentage of fractional shortening (FS%) was calculated by measuring the percentage change in left ventricular dimension to estimate left ventricular function [11]. The LA/Ao was measured in the right parasternal short-axis view in early diastole [2]. The transmitral early diastolic flow (MV E) and tricuspid regurgitant flow velocities were measured in the left apical four-chamber view [11]. The pressure gradient between right ventricle and right atrium or estimated pulmonary arterial pressure (PAP) was calculated from the tricuspid regurgitant flow velocity using the simplified Bernoulli equation (pressure gradient = 4 × velocity [m/s]^2^). Figure 1 shows echocardiographic findings of a dog with PH secondary to DMVD stage C.

### 2.3. Parameters for risk factor analysis

The day of echocardiographic diagnosis of PH secondary to DMVD stage C was defined as the first day of a visit. Echocardiographic parameters evaluated in this study included FS%, LA:Ao, MV E velocity and normalized left ventricular internal diameter in systole (LVIDSN) and in diastole (LVIDDN), according to the Cornell allometric scale method [10]. Information about the dogs was recorded, including age, sex, breed, body weight, the presence of syncope, heart rate, systolic blood pressure, the manifestation of left-sided or biventricular heart failure, radiographic and echocardiographic findings, the presence of left or right heart enlargement, probability of PH, cardiac medical treatments, death-alive status and date of death.

### 2.4. Statistical analysis

Statistical analysis was performed using a commercial statistical programmed (IBM SPSS 22, USA). Descriptive statistical analysis was used to identify population characteristics. Univariable Cox regression was used for the hazard of death. The Kaplan-Meier survival curve was applied for survival analysis. The log-rank test was used to compare differences between survival curves. *P < 0.05* was considered statistically significant. Survival time was expressed as median and 95% confidence interval (CI). Data from live dogs at the end of the study were censored.

## 3. Results

A total of 37 dogs with PH secondary to DMVD stage C were included in this study. The median survival time of 37 dogs with PH secondary to DMVD stage C was 368 days; 95% CI 214.5–584 days. Descriptive data of the dogs recruited for the study, as well as echocardiographic results and medications are presented in Table 1. Data were expressed as medians and interquartile range. There were 17/37 males (45.9%), 13 intact and 4 neutered males, and 20/37 females (54.1%), eight intact and 12 neutered females. The breeds of PH secondary to DMVD stage C dogs in this study were divided into 2 groups. 75.7% (28/37) were purebred dogs and 24.3% (9/37) were mixed breeds. 27% (10/37) were poodles and 73% (27/37) were other breeds including mixed breed (24.3%, 9/37), Chihuahua (21.6%, 8/37), Shih tzu (10.8%, 4/37), Pomeranian (5.4%, 2/37), Miniature pinscher (5.4%, 2/37), Thai Ridgeback (2.7%, 1/37) and Yorkshire (2.7%, 1/37). Thirty-two of 37 dogs (86.5%) had syncope. Seven dogs had signs of right ventricular failure. Five dogs had ascites, and four dogs had pleural effusion. Three dogs had both ascites and pleural effusion. Twenty-three dogs died during the study period and 14 dogs were alive at the end of the study. The age of onset ranged from 7 to 17 years. The dogs received pimobendan (89.19%, 33/37), benazepril (10.81%; 4/37), enalapril (13.51%; 5/37), ramipril (64.86%, 24/37), furosemide (100%, 37/37), spironolactone (21.62; 8/37) and sildenafil. The median daily dose of each medication is summarized in Table 1.

**Table 1.** Descriptive data of dogs with pulmonary hypertension secondary to degenerative mitral valve disease recruited in the study

Category	Median	Interquartile range
Survival time (days)	368	214.5–584
Age (years)	12	10–14
Weight (kg)	4.7	4–6.7
Heart rate (bpm)	142	130.5–175.5
Blood pressure (mmHg)	134	112–154.5
VHS	11.9	11.15–12.73
Echocardiography		
Fractional shortening (%)	50.42	41.43–59.59
LA:Ao	1.87	1.76–1.98
MV E velocity (cm/s)	142.75	128.31–149.35
LVIDSN (mm)	0.99	0.76–1.03
LVIDDN (mm)	1.87	1.72–2.21
Medication (mg/kg/d)		
Pimobendan	0.6	0.47–0.75
Furosemide	3.14	2.38–3.55
Spironolactone	2.25	1.41–3.28
Benazepril	0.39	0.35–0.40
Enalapril	0.74	0.65–1.06
Ramipril	0.14	0.12–0.19
Sildenafil	1.9	1–5.17

VHS, Vertebral Heart Scale; LA:Ao, the ratio of left atrial dimension to the aortic annulus dimension; MV E velocity, Peak velocity of early diastolic transmitral flow; LVIDSN, normalized left ventricular internal diameter in systole; LVIDDN, normalized left ventricular internal diameter in diastole; ACEi, angiotensin converting enzyme inhibitor; mg/kg/d, milligram per kilogram per day

Summary comparisons of median survival time of PH secondary to DMVD stage C dogs in different categories are shown in Table 2. Mixed breed, VHS >11.5, the presence of right heart enlargement and ascites, and a high probability of PH affected survival time of PH secondary to DMVD stage C dogs (Table 2). Other parameters including age, breed (poodle and others), sex, syncope, heart rate, blood pressure, VHS, fractional shortening, LA:Ao, MV E velocity, LVIDSN, LVIDDN, and sildenafil medication did not affect survival time.

**Table 2.** The percentage of dogs with pulmonary hypertension secondary to degenerative mitral valve disease categorized in comparison to the median survival time

Parameter	n	Category	Number (%)	P value	Median	95%CI
Age (years)	37	>7	34 (91.9)		453	301–605
		≤ 7	3 (8.1)	0.325	-	-
Breed	37	Mixed	9 (24.3)		303	100–506
		Pure	28 (75.7)	0.009*	610	377–843
		Poodle	10 (27.0)		652	429–875
		Others	27 (73.0)	0.055	368	261–475
Sex	37	Male	17 (45.9)	0.133	621	219–1023
		Female	20 (54.1)		368	137–599
Syncope	37	Presence	32 (86.5)	0.322	263	221–305
		Absence	5 (13.5)		502	242–762
Heart rate (bpm)**	34	>180	6 (17.6)		379	199–595
		<180	28 (82.4)	0.812	610	329–891
Blood pressure (mmHg)**	29	>160	6 (20.7)		363	148–578
		<160	23 (79.3)	0.32	453	186–720
VHS**	31	>11.5	23 (74.2)		368	257–479
		10.5–11.5	8 (25.8)	0.012*	1238	0
Right heart enlargement	37	Presence	28 (75.7)	<0.0001*	236	84–388
		Absence	9 (24.3)		621	443–799
Ascites	37	Presence	32 (86.5)	0.016*	303	17–589
		Absence	5 (13.5)		601	369–851
Echocardiography						
Fractional shortening (%)	37	>50	18 (48.6)		453	214–692
		<50	19 (51.4)	0.419	610	199–1021
LA:Ao**	31	>1.9	21 (67.7)		379	249–508
		<1.9	10 (32.3)	0.436	-	-
MV E velocity (cm/s)**	31	>150	14 (45.2)		453	152–754
		<150	17 (54.8)	0.769	303	0
LVIDSN (mm)	37	>1	17 (45.9)		397	132–662
		≤1	20 (54.1)	0.833	453	185–721
LVIDDN (mm)	37	>2	18 (48.6)		368	335–401
		≤2	19 (51.4)	0.246	-	-
Probability of PH	37	High	11 (29.7)		253	122–384
		Intermediate	26 (70.3)	0.047*	610	473–747
Medication						
Sildenafil	37	Without	22 (59.5)	0.977	453	298–608
		With	15 (40.5)		610	279–941

VHS, Vertebral Heart Scale; LA:Ao, the ratio of left atrial dimension to the aortic annulus dimension; MV E velocity, Peak velocity of early diastolic transmitral flow; LVIDSN, normalized left ventricular internal diameter in systole; LVIDDN, normalized left ventricular internal diameter in diastole; ACEi, angiotensin converting enzyme inhibitor

* *P < 0.05* indicates statistically significant

** Parameters that were not completed in total of 37 dogs due to lack of data

**Figure 2.**
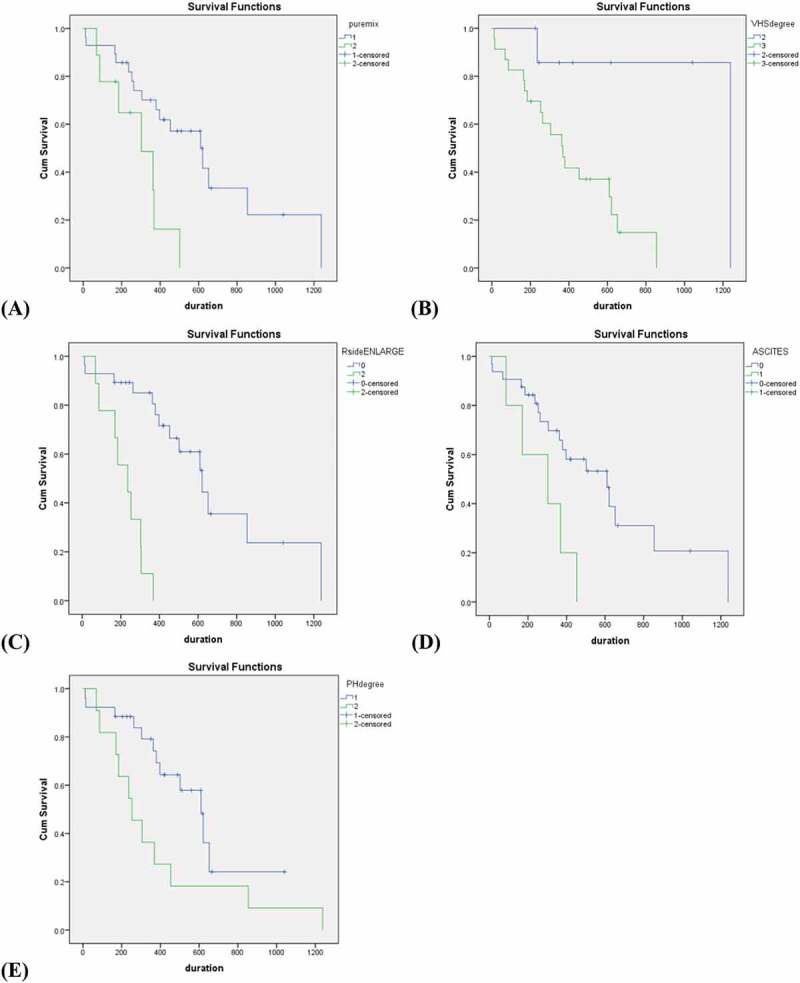
Kaplan-Meier survival curves illustrate survival time for dogs with PH secondary to DMVD stage C in various factors. A, purebred dogs (blue line) and mixed breed dogs (green line) (*p = *0.009). B, the group of dogs with VHS 10.5–11.5 (blue line) and VHS > 11.5 (green line) (*p* = 0.012). C, dogs without presence of right heart enlargement (blue line) and with presence of right heart enlargement (green line) (*p* < 0.0001). D, dogs without presence of ascites (blue line) and with the presence of ascites (green line) (*p* = 0.016). E, dogs with intermediate probability of PH (blue line) and high probability of PH (green line) (*p* = 0.047).

The median survival time of PH secondary to DMVD stage C dogs, between purebred and mixed breeds, was significantly different (*p* = 0.009) (Figure 2 (A), Table 2). Dogs with VHS >11.5 (*p* = 0.012; Figure 2 (B), Table 2), right heart enlargement (*p* = <0.0001; Figure 2 **(C)**, Table 2) and the presence of ascites (*p* = 0.016; Figure 2 (D), Table 2) had a shorter survival time than those without. In addition, the survival time of DMVD dogs with intermediate and high probability of PH was significantly different (*p* = 0.047) (Figure 2 (E), Table 2).

The Cox-regression analysis of numerical and categorical data is summarized in Table 3. Mixed-breed dogs and dogs with VHS >11.5, the presence of right heart enlargement and the presence of ascites were associated with an increased hazard of death (Table 3).

**Table 3.** The result of the Cox-regression analysis in numerical data and categorical data for the hazard of death

Parameter			Category	Hazard ratio		95%CI	P value
Numerical data							
Age (Years)				1.146		0.970–1.353	0.110
Heart rate (bpm)				1.006		0.990–1.022	0.460
Blood pressure (mmHg)				1.001		0.983–1.020	0.876
VHS				1.664		1.084–2.555	0.020*
Echocardiography							
Fractional shortening (%)				1.015		0.966–1.066	0.564
LA:Ao				2.099		0.882–4.995	0.094
MV E velocity (cm/s)				0.999		0.985–1.013	0.874
LVIDSN (mm)				0.815		0.163–4.075	0.803
LVIDDN (mm)				1.477		0.349–6.253	0.596
Categorical data							
Age (years)			>7 and ≤7	2.644		0.353–19.785	0.344
Breed			Mixed and Pure	3.453		1.298–9.181	0.013*
Sex			Male and Female	0.517		0.215–1.241	0.140
Syncope			Presence and absence	1.859		0.534–6.471	0.330
Heart rate (bpm)			>180 and <180	1.166		0.328–4.150	0.812
Blood pressure (mmHg)			>160 and <160	1.686		0.596–4.769	0.325
VHS			>11.5	5.599		0.278–24.522	0.022*
			10.5–11.5				
Right side enlargement			Presence and absence	10		3.193–31.506	<0.0001*
Ascites			Presence and absence	3.397		1.185–9.733	0.023*
Echocardiography							
Fractional shortening (%)		>50 and <50		1.412		0.609–3.277	0.421
LA:Ao		>1.9 and <1.9		1.647		0.463–5.864	0.441
MV E velocity (cm/s)		>150 and <150		0.869		0.341–2.216	0.769
LVIDSN (mm)		>1 and ≤1		0.913		0.393–2.122	0.833
LVIDDN (mm)		>2 and ≤2		1.708		0.684–4.266	0.251
Probability of PH		high and Intermediate		2.333		0.987–5.513	0.053
Medication							
Sildenafil		With and without		0.988		0.423–2.305	0.977

VHS, Vertebral Heart Scale; LA:Ao, the ratio of left atrial dimension to the aortic annulus dimension; MV E velocity, Peak velocity of early diastolic transmitral flow; LVIDSN, normalized left ventricular internal diameter in systole; LVIDDN, normalized left ventricular internal diameter in diastole; ACEi, angiotensin converting enzyme inhibitor

* *P < 0.05* indicates statistically significant

## 4. Discussion

The main findings of this study were: 1) the median survival time of dogs with PH secondary to DMVD stage C was 368 days, 2) mixed-breed dogs and dogs with greater VHS, right heart enlargement, ascites and high probability of PH had shorter survival time of dogs affected by PH secondary to DMVD stage C, 3) mixed-breed dogs and dogs with right heart enlargement and ascites had an increased hazard of death.

The results of this study showed that the median survival time in dogs with PH secondary to DMVD stage C was 368 days. The median survival time from this study was longer than that in dogs with advanced heart failure secondary to DMVD, which was 281 days [12], and in dogs with PH secondary to respiratory disease, which was 276 days [13]. A previous study showed that the median survival time of stage C DMVD dogs was 491 days and that PH dogs secondary to DMVD stage B2 and C had a median survival time of 456 days [5]. However, survival times from different studies are not comparable because various factors such as medications and owner care may influence survival time.

The median age of dogs with PH secondary to DMVD stage C in this study was 12 years (range 10–14 years). This result is in consistent with a previous study suggesting that the prevalence of DMVD in small-breed dogs increases significantly with age (mean 10.7 ± 2.7 years) [5]. However, according to the results of this study, age did not affect the survival time of dogs with PH secondary to DMVD stage C.

Previous studies have shown that small-breed dogs, such as Cavalier King Charles Spaniels (CKCS), Poodles, Chihuahuas and Shih Tzus, are predisposed to development of DMVD [2,14]. A previous study reported that CKCS and other purebred dogs were at higher risk for cardiac-related death than mixed-breed dogs [14]; however, the median survival time of purebred dogs in this study was longer than that of mixed-breed dogs. In addition, mixed-breed dogs had a 3.5 times higher hazard of death than purebred dogs.

Dogs with an increased VHS >11.5 tended to have a shorter survival time and a 5.6 times increased hazard of death compared with dogs with a VHS < 11.5. An increase in heart size is associated with an increase in disease severity. This finding is consistent with a previous study reporting that heart enlargement was associated with decreased survival time and increased risk of death in dogs with DMVD [7]. The presence of right heart enlargement was also associated with a shorter survival time and a 10 times higher hazard of death than in dogs without right heart enlargement in the present study. A previous study has also demonstrated the association between right atrial size and decreased right ventricular function in dogs with PH from various causes [15]. Visser et al [15] also mentioned that enlargement of the right heart is common in dogs with PH. When PH is maintained, the right ventricle has to work harder against the increased pulmonary pressure, and overload of the right ventricle leads to cardiac remodelling and right ventricular dysfunction. Eventually, right-sided heart failure occurs [16,17]. This leads to fluid accumulation in the abdominal, pleural and/or pericardial cavities [17], which can sometimes be controlled with medication [18]. This study showed that dogs with ascites have a shorter survival time and a 3.4 times higher hazard of death than dogs without ascites. These results suggest that ascites may be used as an indicator of prognosis in dogs with PH secondary to DMVD stage C. More than 80% of dogs in this study had signs of syncope, a common clinical sign of dogs with PH [4]. However, presence of syncope did not affect the survival time of dogs with PH secondary to DMVD stage C in the present study.

The medical treatment of PH secondary to DMVD aims to lower the systolic pulmonary artery pressure (sPAP). Previous studies showed that median survival time of dogs treated with sildenafil was 91 days [19] and 8 to more than 734 days 20. Medications include platelet-derived growth factor inhibitors, prostacyclin analogues, endothelin antagonists, and phosphodiesterase-5 inhibitors (PDE-5is) [16,20]. Sildenafil is a PDE-5is that has a selective vasodilatory effect on pulmonary vessels [16]. Previous studies have shown that sildenafil can decrease sPAP in PH due to respiratory and cardiovascular diseases [16,20,21]. According to the results of this study, the median survival time of dogs treated with sildenafil was 453 days and without sildenafil was 610 days. Therefore, sildenafil had no effect on the survival time of dogs with PH secondary to DMVD in this study.

Although the study was carefully prepared, data from some dogs were not collected because of the limited information that could be obtained from their owners. This limitation resulted from the retrospective design of the study. Because the missing data were different for each parameter, multivariate regression analysis could not be performed. In addition, data of complete blood counts and blood chemistry could not be analysed in this study because the variation of blood collection time in each dog.

## 5. Conclusion

This study reports an estimated survival time of PH secondary to DMVD stage C dogs in Thailand. Mixed breed, VHS>11.5, the presence of right heart enlargement, ascites and high probability of PH were related to a shorter survival time in dogs with PH secondary to DMVD stage C. Therefore, these parameters should be included in the diagnostic investigation and prognosis of DMVD dogs with PH.
